# Experiences of Australian podiatrists working through the 2020 coronavirus (COVID-19) pandemic: an online survey

**DOI:** 10.1186/s13047-021-00449-9

**Published:** 2021-02-03

**Authors:** Cylie M. Williams, Anna Couch, Terry Haines, Hylton B. Menz

**Affiliations:** 1 Peninsula Health, Allied Health, Frankston, VIC 3199 Australia; 2 School of Primary and Allied Health Care, Monash University, Moorooduc Hwy, Frankston, VIC 3199 Australia; 3 Discipline of Podiatry, School of Allied Health, Human Services and Sport, La Trobe University, Melbourne, Victoria 3086 Australia

## Abstract

**Background:**

On the 19th of January, 2020, the Chief Medical Officer of Australia issued a statement about a novel coronavirus, or SARS-CoV-2. Since this date, there have been variable jurisdictional responses, including lockdowns, and restrictions on podiatry practice. This study aimed to describe impacts of the SARS-CoV-2 pandemic on the podiatry profession in Australia.

**Methods:**

This was a cross sectional study of Australian podiatrists using demographic data collected between 2017 and 2020, and pandemic-related question responses collected between 30th March and 31st August, 2020. Data were collected online and participants described their work settings, patient funding types, business decisions and impacts, and information sources used to guide practice decisions during this time-period. Inductive thematic analysis was used to analyse open-ended questions about their practice impact of SARS-CoV-2.

**Results:**

There were 732 survey responses, with 465 Australian podiatrists or podiatric surgeons providing responses describing pandemic impact. From these responses, 223 (49% of 453) podiatrists reported no supply issues, or having adequate supplies for the foreseeable future with personal protective equipment (PPE) or consumables to support effective infection prevention and control. The most frequent responses about employment, or hours of work, impact were reported in the various categories of “business as usual” (*n* = 312, 67%). Participants described most frequently using the local state and territory Department of Health websites (*n* = 347, 75%), and the Australian Podiatry Association (*n* = 334, 72%) to make decisions about their business. Overarching themes which resounded through open-ended comments was that working through the pandemic was likened to a marathon, and not a sprint. Themes were: (i) commitment to do this, (ii) it’s all in the plan, but not everything goes to plan, (iii) my support team must be part of getting through it, (iv) road blocks happen, and (v) nothing is easy, what’s next?

**Conclusion:**

Podiatrists in Australia reported variable pandemic impact on their business decisions, PPE stores, and their valued sources of information. Podiatrists also described their “marathon” journey through the pandemic to date, with quotes describing their challenges and highlights. Describing these experiences should provide key learnings for future workforce challenges, should further restrictions come into place.

**Supplementary Information:**

The online version contains supplementary material available at 10.1186/s13047-021-00449-9.

## Background

**Fig. 1:**
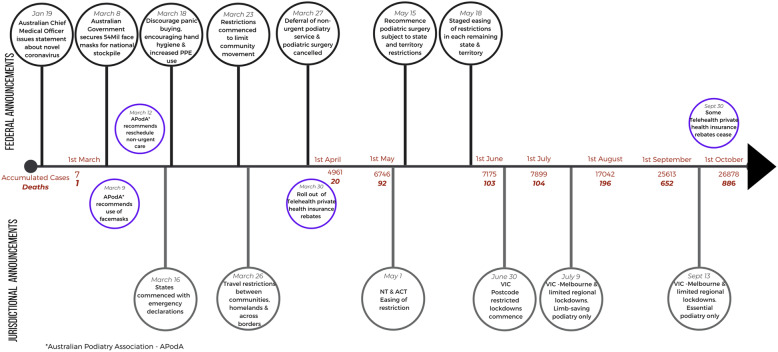
Timeline of Directives Impacting podiatry through Australia From January to October, 2020

On the 19th of January, 2020, the Chief Medical Officer of Australia issued a statement about a novel coronavirus or SARS-CoV-2, internationally reported to the World Health Organisation [1]. At that stage, very little was known about its transmission, severity and risk factors. The first set of restrictions aiming to slow transmission of this virus were announced in Australia on the 15th of March [2]. These restrictions were rapidly tightened until there were variable state and territory lockdowns or public movement restrictions in place by the end of March. It is from this date that recommendations for triaging and changes in requirements for who could seek podiatry care commenced across the country (Fig. 1). Also at this date, there were 3966 cumulative cases and 16 deaths in Australia from complications relating to the virus, against a backdrop of 62,308 confirmed cases and 3390 deaths globally [3].

Since this time, much has been learned about SARS-CoV-2 and coronavirus disease, also known as COVID-19. COVID-19 is an infectious pneumonia with classical viral symptoms such as fever, muscle soreness with fatigue, shortness of breath and cough [4]. Less common symptoms include altered taste and smell, and vascular-related skin lesions [5]. SARS-CoV-2 is transmitted through aerosolised droplets and is recognised as highly infectious [6]. There is variable mortality from the infection, but those who are immunocompromised or have a chronic disease are most susceptible to death, with mortality rates for older adults as high as 7.8% [7].

Although the Australian government established the National COVID-19 Coordination Commission to coordinate the national response to the pandemic, infection prevention and control responses varied in each state and territory, some of which resulted in lowering the spread of the virus in the community. This first wave of control early March involved recommendations to health workers to minimise face to face service delivery, increase social distancing requirements in their clinical settings, and increase the amount of personal protective equipment (PPE) worn with all patients [8]. However, there were a number of government recommendations and infection prevention and control breaches since March 2020, resulting in a second wave in Victoria and continued state of emergency [9]. One of the internationally strictest lockdowns and movement restrictions ensued in metropolitan Victoria in order to minimise transmission across the city and state. As of the beginning of October, Australia has seen 27,121 confirmed cases and 893 deaths, compared to a global cumulative case number of > 34.5 million, and over 1 million deaths [3].

In Australia, an estimated 60% of the podiatry profession are located in two states (Victoria and New South Wales) [10] which also had the greatest number of community SARS-CoV-2 transmissions to date [11]. Many international recommendations for podiatry triage and risk identification were made during the first three months of the pandemic, however none of these were contextualised to the Australian health system or private practice settings [12, 13]. Instead, government recommendations were made on the provision of essential podiatry services only. During this time also, research about SARS-CoV-2 and COVID-19 primarily focused on epidemiology, aiming to understand the virus, develop vaccines, reduce transmission and decrease mortality. There has been little investigation of the impact of the pandemic on the allied health workforce, and what has been published has been primarily editorial in nature [14, 15]. Workforce research has particularly focused on roles traditionally considered “frontline” such as medical professionals, nurses and paramedics, with research focused on psychological impact and little on change in employment or service delivery [16–18].

Our primary aim of this study was to describe the impact of the SARS-CoV-2 pandemic in Australian podiatry settings. Our secondary aims included (i) describing the podiatry workforce decisions of closure and employment, (ii) identifying where podiatrists sought their information to make practice closure and environmental change decisions, and (iii) to synthesise podiatrists’ clinical lived experience during the pandemic.

## Methods

### Design

This was a cross sectional study of Australian podiatrists using demographic data collected between 2017 and 2020. Each of the four data waves were open for approximately 6 months each year and pandemic-related question responses were added to the fourth wave and collected between 30th March and 31st August, 2020, at the height of Australian practice-based restrictions. Approval was given by the Human Research Ethics Committees of Monash University (19959). The CHERRIES (Checklist for Reporting Results of Internet E-Surveys) guided the reporting of collected data [19].

#### Participants and setting

Australian podiatrists and podiatric surgeons were invited to participate in all four waves of the research project titled: Podiatrists in Australia: Investigating Graduate Employment (PAIGE). At the time of the fourth wave survey closure, there were an estimated 5429 podiatrists and 36 podiatric surgeons registered as practicing in Australia [10]. Participants were recruited each year through promotion of the survey at Australian conferences, social media (Facebook, Twitter, LinkedIn and Instagram) and through targeted emails from peak bodies such as the Australian Podiatry Association and Australasian College of Podiatric Surgeons. Participants were also directly invited via email to complete each wave of the survey if they had completed a survey in a previous year. There were competition-based incentives through the recruitment processes, the most common being a $100 (AUD) voucher for up to 10 participants to be used from the Australian Podiatry Association for educational purposes. The winners were drawn in a way that survey responses could not be linked.

### Measurements

The PAIGE study methodology and survey tools were based on the Medicine in Australia: Balancing Employment and Life (MABEL) study [20]. The primary aim of the PAIGE study was to investigate intrinsic and extrinsic labour decisions. Questions were modelled on the MABEL study with similar wording for demographic data collection, in addition to measurement of constructs impacting on labour decisions such as job satisfaction (all waves), earnings (Wave 1), impact on family (Wave 1), workplace setting (all waves), mental health (Waves 2, 3 and 4) and attributes impacting on life-long learning (Wave 4). The fourth year of PAIGE data collection coincided with the SARS-CoV-2 pandemic. Questions were added to include the impact of the pandemic on PPE and consumables, business decisions on closure and current working situation, information sources for making business decisions and the perceived helpfulness (4-point Likert scale with 0 - No help at all to 4 - Very helpful) of these sources, and a free text box inviting commentary on the impact of the pandemic on individual and practice circumstances. The full Wave 4 survey is provided as Supplementary File 1.

Demographic variables extracted from any wave for this present survey included:

(i) Age in 2020 (years)
(ii) Recency of practice in 2020 (years)
(iii) Practice jurisdiction (State or Territory)
(iv) Primary workplace setting
(v) Business relationship with main workplace
(vi) Number of working locations
(vii) Percentage of clinical load including bulk billed Medicare chronic disease management plan
(viii) Percentage of clinical load involving telehealth consultations
(ix) Percentage of clinical load assessing or treating patients who are National Disability Insurance Scheme (NDIS) participants
(x) Percentage of working week involving home visits

### Procedure

Each wave of survey data were collected online via Qualtrics® software (Qualtrics, Provo, UT, USA) [21] and subsequent waves linked through a self-generated code. Participants were asked to identify past responses which dictated question logic. If it was the participant’s first time completing (in any year), they were asked questions relating to job satisfaction, location, training, and questions about their podiatry practice such as setting, hours of work, hours in spent face to face care. Where a podiatrist indicated they had previously participated, only demographics such as gender, year of both and year of graduation were shown in addition to new questions. Forced or requested responses were used to minimise missing data, but podiatrists could close and exit the survey at any time. Cookies were used to allow responses to be saved up to 4 hours within partial completion. Qualtrics® routinely collects Internet Protocol (IP) addresses as part of the de-identified metadata in the survey response. IPs were only viewed and used as a last resort to match data where other linking variables were incomplete.

### Analysis

Data were initially cleaned and responses removed if age or gender was unanswered or unable to be matched to previous waves. Partial responses were retained within pandemic response questions if at least one demographic wave question set, and at least one pandemic question was completed. Data were analysed in Stata 15 (StataCorp, College Station, TX, USA) [22]. Descriptive statistics were initially used to report on each variable of interest for the entire cohort where there were greater than 5 individual responses for that item. Less than 5 responses in any one demographic item were generally not reported due to the potential identifiable nature of the variable. Descriptive statistics were then grouped by states and territories due to the variable jurisdictional responses to SARS-CoV-2 positive cases and community transmission. The Australian Federal Government announced a number of financial support packages for individuals and businesses on the 30th of March, 2020. Survey questions relating to pandemic impact were collected from the same day, therefore no date stratification of responses was undertaken.

Inductive thematic analysis of the single open text question was undertaken by hand. This method of analysis allowed for statements and comments to be individually considered and these statements used for theme generation [23]. Originally, the statements were grouped against concepts and then during concept review, meaningful themes were developed. There were a variety in the length of statements by participants. Even if the statement was one sentence, it was grouped where possible. This grouping took an iterative approach, whereby if a new concept or theme developed, earlier statements were recoded.

The data were initially analysed by a single researcher (CW). To reduce individual bias, the themes and individual statements were independently reviewed by the second author (AC) and disagreements were resolved by discussion. Reflexivity was acknowledged as a concept that introduces personal bias into research [24]. Authors analysing qualitative data acknowledged their different individual experiences working within public and private podiatry settings during the pandemic, and how these different experiences may have influenced the analysis.

## Results

### Participant characteristics

There were 732 responses to the survey, with 465 (9% of 5465 registered practicing podiatrists and podiatric surgeons [10]) responses containing data enabling descriptive analysis of workforce impact from SARS-CoV-2. The results from here on relate to the 465 podiatrists’ responses. As podiatrists were able to exit the survey at any time; any partial pandemic related responses were retained and reported. Table 1 displays a breakdown of the 465 podiatrists’ demographics, work setting and practice profile according to total responses, and responses from each state and territory. This is one of the first studies to explore the levels of podiatrists engaging with telehealth in general care, up to (*n* = 96) 25% of responding podiatrists reported utilising telehealth during this time.

**Table 1:** Demographics of participants presented with their mean (SD), frequency (%), *median (IQR)* and *range*

	Total responses *n* = 465 (100%)	Victoria *n* = 221 (50%)	New South Wales *n* = 81 (18%)	Queensland *n* = 58 (13%)	South Australia *n* = 39 (9%)	NT/TAS/ACT* n = 23 (5%)	Western Australia *n* = 24 (5%)
**Age** (Years)	*36 (30,46), 22–73*	*34 (29,43) 23–62*	*42 (35,49), 23–73*	*37 (29,46), 22–64*	*36 (29,50), 23–61*	*34 (30,43), 23–61*	*46 (31,47.5), 24–62*
**Gender** (Female)	341 (73%)	151 (68%)	55 (68%)	38 (66%)	29 (74%)	12 (52%)	18 (75%)
**Recency**	***N*** **= 464**						
0–5 years	113 (24%)	42 (19%)	16 (20%)	19 (33%)	6 (15%)	6 (26%)	4 (16%)
6–10 years	108 (23%)	71 (32%)	18 (22%)	11 (19%)	9 (23%)	6 (26%)	3 (13%)
>10 years	243 (53%)	108 (49%)	47 (58%)	28 (48%)	24 (62%)	11 (48%)	17 (71%
**Primary work setting**	***N*** **= 443**	***n*** **= 218**					
Private practice	299 (67%)	130 (60%)	61 (75%)	45 (78%)	32 (82%)	13 (57%)	18 (75%)
Public health service	136 (3%)	85 (39%)	16 (20%)	13 (22%)	7 (18%)	10 (43%)	5 (21%)
Administration/University	8 (2%)	3 (1%)	4 (5%)	–	–	–	1 (4%)
**Business relationship**	***N*** **= 444**	***n*** **= 219**					
Owner or partner	156 (35%)	64 (29%)	37 (46%)	19 (33%)	20 (51%)	5 (22%)	11 (46%)
Salaried/Contract	283 (64%)	153 (70%	43 (53%)	39 (67%)	17 (44%)	18 (78%)	13 (54%)
Locum/Not working	5 (1%)	2 (1%)	1 (1%)	–	2 (5%)	–	–
**Working locations**	***N*** **= 428**	***n*** **= 216**					**n = 23**
1	181 (42%)	86 (40%)	35 (44%)	23 (40%)	14 (36%)	10 (43%)	13 (54%)
2–3	194 (45%)	95 (44%)	36 (44%)	27 (46%)	20 (51%)	8 (35%)	8 (33%)
> 3	53 (12%)	35 (16%)	10 (12%)	8 (14%)	5 (13%)	5 (22%)	2 (13%)
**Medicare caseload **							
0%	337 (72%)	164 (74%)	62 (77%)	35 (60%)	24 (62%)	20 (87%)	17 (71%)
1–50%	42 (10%)	22 (10%)	6 (7%)	7 (12%)	6 (15%)	–	1 (4%)
> 50%	86 (18%)	35 (16%)	13 (16%)	16 (28%)	9 (23%)	3 (13%)	6 (25%)
**Telehealth caseload**	***N*** **= 378**	***n*** **= 173**	***n*** **= 69**	***n*** **= 50**	**n = 34**	**n = 21**	***n*** **= 19**
0%	279 (74%)	115 (66%)	60 (87%)	43 (86%)	28 (82%)	14 (67%)	15 (79%)
1–50%	96 (25%)	56 (32%)	9 (13%)	7 (14%)	5 (15%)	7 (33%)	7 (21%)
> 50%	3 (1%)	2 (1%)	–	–	1 (3%)	–	–
**NDIS caseload**							
0%	301 (64%)	139 (63%)	55 (68%)	39 (67%)	20 (51%)	14 (61%)	19 (79%)
1–50%	163 (35%)	79 (36%)	26 (32%)	19 (33%)	18 (46%)	9 (39%)	5 (21%)
> 50%	1 (1%)	3 (1%)	–	–	1 (3%)	–	–
**Home visits**	***N*** **= 307**	***n*** **= 156**	***n*** **= 57**	***n*** **= 45**	***n*** **= 25**	***n*** **= 10**	**n = 10**
0–50%	287 (93%)	146 (94%)	53 (93%)	41 (91%)	24 (96%)	10 (100%)	10 (100%)
> 50%	20 (7%)	10 (6%)	4 (7%)	4 (9%)	1 (4%)		

### Availability of personal protection equipment and consumables

From these responses, 223 (49% of 453 responses) podiatrists reported no supply issues or having adequate supplies for the foreseeable future with PPE or consumables to support effective infection prevention and control (Table 2). During the data collection timeframe, up to 19% (85 of 453 responses) reported no or limited stock of some PPE components. Of these, the predominant lack of stock was masks, with some also describing lack of disposable gowns or wipes used for disinfecting surfaces.

**Table 2:** Participant responses (frequency (%)) to questions relating to the level of personal protection equipment (PPE) and consumables to undertaken infection prevention and control in their practice setting

	Total responses *N* = 453	Victoria *n* = 211 (47%)	New South Wales n = 81 (18%)	Queensland n = 58 (13%)	South Australia n = 39 (9%)	NT/TAS/ACT* n = 23 (5%)	Western Australia n = 24 (5%)
No PPE or consumable stock supply issues	223 (49%)	112 (53%)	47 (55%)	26 (45%)	17 (44%)	8 (35%)	12 (50%)
< 2 months PPE/hand hygiene products	145 (32%)	68 (32%)	19 (23%)	24 (41%)	14 (36%)	8 (35%)	8 (33%
Limited stock of some PPE components	59 (13%)	27 (13%)	11 (14%)	5 (9%)	4 (10%)	7 (30%)	3 (13%)
No waterless hand hygiene products	3 (1%)	1 (1%)	1 (1%)	–	1 (3%)	–	–
No stock of some PPE components	23 (5%)	3 (1%)	3 (4%)	3 (5%)	3 (7%)	–	1 (4%)

### Employment decisions

“Business as usual” (*n* = 312, 67%) was the most frequent response to the various categories of business or employment conditions during the pandemic (Table 3). Podiatrists also frequently responded to the category “Business as usual, but with developed plans for cessation of service”, and these responses varied across the states. Western Australia had the highest jurisdiction response of those completing the survey (*n* = 6, 25%), however there were overall low responses from the 492 podiatrists within Western Australia [10]. Therefore, this response is unlikely to be truly representative of the total Western Australian podiatry profession. Participants from Victoria (*n* = 43, 19%) and Queensland (*n* = 12, 21%) reported having their hours reduced or ceased during this time with or without pay.

**Table 3:** Participant responses (frequency (%)) to questions relating to closure and employment business decisions. Participants were asked to select the best fit for their primary work setting

	Total responses N = 464	Victoria n = 221 (48%)	New South Wales *n* = 80 (17%)	Queensland n = 58 (13%)	South Australia n = 39 (9%)	NT/TAS/ACT* n = 23 (5%)	Western Australia n = 24 (5%)
Business as usual (Self-employed, business owners, employees)	312 (67%)	145 (66%)	57 (71%)	34 (59%)	35 (90%)	15 (65%)	12 (50%)
Business as usual but with developed plan/s for practice closure	74 (16%)	33 (15%)	13 (16%)	12 (21%)	3 (8%)	5 (22%)	6 (25%)
My employer reduced my hours with paid leave	21 (5%)	13 (6%)	2 (3%)	5 (9%)	–	–	–
My employer reduced my hours, with no paid leave	29 (6%)	14 (6%)	4 (5%)	6 (10%)	1 (2%)	2 (9%)	1 (4%)
I am currently not working	28 (6%)	16 (7%)	4 (5%)	1 (1%)	–	1 (4%)	5 (21%)

*Northern Territory/Tasmania/Australian Capital Territory

### Sources of information and perceived value

Some participants described most frequently using the local state and territory Department of Health websites (*n* = 347, 75%), and the Australian Podiatry Association (*n* = 334, 72%) to make decisions about their health and business (Table 4). These sources were also rated as being the most helpful.

**Table 4:** Participant responses (n = 465) to where they obtained information to guide their health and business decisions (n,%) and perceived helpfulness (Median, IQR)

	Reported use in past 7 days n (%)	Perceived helpfulness Median (IQR)
Department of Health (State/Territory)	347 (75%)	3 (3, 4)
Australian Podiatry Association	334 (72%)	3 (3, 4)
Department of Health (Federal)	291 (63%)	3 (3, 4)
Friends/family (health professionals)	292 (63%)	3 (2, 4)
Facebook (feed or group)	273 (59%)	3 (2, 4)
Local health services (websites, newsletters)	225 (48%)	3 (2, 4)
COVID-19 Government app	214 (46%)	3 (2, 4)
Friends/family (non-health professionals)	209 (45%)	2 (1, 3)
Regulatory bodies	203 (44%)	3 (2, 4)
Twitter	51 (11%)	1 (1, 3)

*Open ended responses to question: “Any other comments on the impact of COVID-19 on your practice”.*

Participants provided rich responses to the open-ended question about the impact of the SARS-CoV-2 pandemic on them and their practice. The overarching theme which resounded through their comments was that the pandemic was likened to a marathon and not a sprint. This analogy resulted in five superordinate themes were generated during the analysis. These were: (i) commitment to the end game and big picture, (ii) it’s all in the plan, but not everything goes to plan, (iii) I need support from my team, but I am also the support crew for others, (iv) road blocks happen, and (v) nothing is easy, what’s next?

#### Theme 1: Commitment to the end game and big picture

Participants described a commitment to public health messaging and having a role as public health advocates. These roles were described as personally positive, but had a negative impact on their practice. These challenges related from perceptions that the government and those making recommendations, not seeing the podiatry profession as important as other professions. It also challenged participants with how to safely provide services in their clinic or aged-care facilities. These comments were particularly focused around concern for the health and safety of people seeking podiatry service. This was highlight by the quote:

*“has reduced patient numbers / income but under the social contract I am in a privileged position to be an essential worker but with this privilege comes responsibility to do what is best for society in this time of crisis which means being more selective in who I see and why I see them” (p60).*

Participants also described additional actions taken to support any employed staff, and people seeking care in their clinic during the various pandemic stages:

*“Have done up an A4 flyer on “How to wear a mask” as I spent a lot of time helping people understand this, after seeing what they were doing with their masks!” (p403).*

#### Theme 2: It’s all in the plan, but not everything goes to plan

Participants described the challenge of setting themselves up with a plan, just like a marathon. They described preparation, educating themselves and then what happens when they hit a roadblock or required a detour to their plan. This planning theme with resultant actions was highlighted in a number of subthemes including (i) I have all the knowledge and skills to keep me and my patients safe, and (ii) I’m off on a detour, but have the skills to get back on path.

While describing (i) I have all the knowledge and skills to keep me and my patient safe, participants described how prepared they felt in some areas as infection prevention and control. They highlighted the additional cleaning load in between people attending their clinic, and the challenge of wearing PPE for longer than usual:

*” I am wearing full PPE gear when I conduct my treatments and am spreading out my appointments so I can do a thorough sanitisation” (*p373).

Despite this being described as core business, participants also described the additional physical and mental health burdens of having to continually use information to guide decisions, wearing PPE and cleaning:

*“Physical work load has decreased with COVID-19 but arriving home totally exhausted” (p372).*

And:

“*… staggered appointments, temperature and oxymeter checks, masks and gloves for reception, opening and closing door for patients, hand sanitiser, posters, following guidelines closely, reassuring patients” (p96).*

Participants also described career impact resulting from workplace responses under the subordinate theme: (ii) I’m off on a detour, but have the skills to get back on path. Within this subtheme, there were alternate tasks performed by some participants as part of their employment. These included redeployment into contact tracing teams, COVID-19 testing, ordering PPE for their health service, and being on notice for redeployment into hospital teams without traditional podiatry roles, or utilising skills and knowledge in private practice to support patient care:

*“The infection control guidelines already in place prior to Covid have been entrenched in our clinical behaviour so as a Podiatrist, I feel we were able to adapt to the few extra duties like educating patients to hand sanitise prior to sitting and taking temperatures” (p354).*

#### Theme 3: I need support from my team, but I am also the support crew for others

Participants described the challenges and supportive roles their teams and other people played during this journey. Subthemes arose relating to rapidly evolving environmental challenges such as (i) information overload, and how this overload was managed, and a second subtheme relating to support teams and their concern for both the future health of people seeking care with (ii) will people still need me to support them as their health professional?

Participants initially described banding together with colleagues and staff to navigate the rapidly evolving health landscape and recommendations. Reflections including positive communication, how things could have been done better, or when things did not go to plan:

*“We are having a daily zoom meeting with our CEO, board members and several hundred employees at a time, every day. We have been updated on a daily basis, and feel very comfortable with our current working arrangements to continue practising, would we consider an essential health service …*” *(p13).*

And:

*“The not knowing what to do and how to plan ahead when things change every day. The rapidly changing work environment has been a big challenge and we have made it through and I feel most staff are happy now, but it has been VERY HARD!” (p452).*

Participants also described their concern for the health and wellbeing people who regularly seek their support for foot health. These responses included increased cancellations, rescheduling of regular appointments and then subsequent complications developing or people being confused as to when they can seek podiatry care due to rapidly changing recommendations from authorities:

*“At initial shutdown business was affected with a decrease in clients wanting to attend. As time has gone on clients are wanting to continue their treatment” (p41).*

And:

*“Patient dissatisfaction at new 6 week DHS [Department of Health Services] restrictions to work. Very stressful to determine urgency of a small portion of my patients. Some patients view their condition as urgent while I may disagree. This brings increased stress in already difficult times. I want to do right by my patients and duty of care and also adhere to DHS guidelines” (p419).*

#### Theme 4: roadblocks happen

Similar to the marathon, the theme of roadblocks arising at different points in the journey also arose within participant responses. Participants described their experiences depending on their state and territory restrictions in relation to their timing of survey completion. Their responses were grouped into a number of roadblock subthemes including: (i) well, that was a speed bump, through to (ii) hitting the wall.

The SARS-CoV-2 pandemic had vastly different impacts across Australia and this difference was highlighted, particularly in these two subthemes. Participants described some initial challenges during the pandemic relating to a short but dramatic business downturn which turned around with lower local community acquired infections. This experience was highlighted through economic challenges reported by both business owners and employee participants:

*“I remain busy, but have had an increase in DNA [did not attend] and last minute cancellations which has put strain on the cashflow. But not 30%, so am considering options to remain financially viable” (p104).*

And:

*“Devastating. Haven’t taken income since March to try to keep staff employed. Loans on hold, Job Keeper. Limited patients. OR [Operating Room] work very limited” (p23).*

Other participants described (ii) hitting the wall, and not being sure how to move on from the challenge. This included accumulative impacts from recent bushfire disasters in large parts of Australia [25]. Participants who were also employers described the pressure to look after themselves, their families, their staff and their patients, and it having a negative impact on their own mental health. Location challenges and jurisdictional border restrictions also resulted in unexpectedly having to cease service delivery:

*“It was stressful at the peak and I had to close and take time out until mentally and physically equipped to provide optimum care” (p16).*

And:

*“We have been affected by the NSW/Victorian border closure with some of my colleagues living outside the bubble and unable to attend work as usual” (p86).*

#### Theme 5: Nothing is easy, what’s next?

Participants described seeing an end in sight, or feeling like they are on the other side of challenges. They described learning about practice behaviours and enhancements as they made plans to move forward. Some also described increase in business or additional opportunities as restrictions were locally eased, which made it easier to re-establish or continue with their practice. Others described using it as a way to increase self-management strategies during telehealth:

*“As a subcontractor in a private clinic my patient load has decreased by approx 50%, however as a sole trader mobile podiatrist travelling to people’s homes who may be self-isolating my business has increased by approx 20%” (p150).*

And:

*“Conversion to telehealth where appropriate. I have found COVID-19 and restrictions has required patients to improve their self-management skills and take more ownership of their foot health” (p166).*

## Discussion

There is very little known about the impact of the SARS-CoV-2 pandemic on allied health care and private practice business decisions. This present study reports the first known data collected about the impact of SARS-CoV-2 on the podiatry profession. Other research has primarily focused on opportunities and challenges of hospital based service delivery [14], and have provided recommendations for triage through variable staging of restrictions to delivery of health care in public or hospital settings [26] and reflections of the different public health restrictions and guidance for podiatry services primarily within the United Kingdom [27].

One important finding within this study was the variable disruption to service as a result of recommendations, and the resilience podiatrists demonstrated during this historical event. Despite the differing jurisdictional recommendations of service continuance, service delivery appeared minimally impacted. Participants often reported these impacts as a direct response to the business decisions at the time of answering the survey and that these varied with time. It was not pre-planned to stratify responses into months or according to government restricted service timeframes, however with larger responses, this may have been an approach to understand how government directives impacted triage decisions and workforce employment. Given there were between 10 and 14 weeks in Victoria where podiatrists were advised to provide limb saving podiatry services only [28], it should be expected that responses reflected some loss of hours and work over that time, as many podiatrists in private practice may not commonly provide treatment to patients requiring that level of urgent care. Alternatively, podiatrists may have adapted their clinics to telehealth or alternative service provisions, or even decided based on their patient’s foot health and well-being that they could justify their patients meeting the criteria set by the government during any restricted service timeframes.

Due to the novel nature of this study, comparisons to other allied health professions are not possible. It is also unclear to what extent these findings could be generalised outside of the Australian healthcare context or for international podiatry practice. Australian primary care practice nurses reported experiencing greater loss of hours during the peaks of restricted practice, and similar to podiatrists, rarely experienced limited access to the required PPE in the workplace [16]. These important findings highlight exposure to vulnerability that many small businesses faced, where models of care are not fully reliant on billable government Medicare funds, or funding primarily accessed by medical practitioners. It also highlighted the success Australia had relating to access of PPE, with minimal impact of most PPE stocks, at least to the appropriate level within community based private practice settings. PPE stocks appears to be an ongoing challenge facing international colleagues, with recent reports of as high as 39.7% of health care workers reporting reuse or inadequate supply of appropriate PPE in the United Kingdom and United States [29]. We had limited numbers of podiatrists who responded working from public hospitals, and it is unknown how many provided care to COVID-19 positive patients, so we are unable to make any comparisons on the availability of the higher levels of PPE required in these settings.

This is also one of the first studies to explore the use of telehealth for general conditions presenting to podiatry. Targeted telehealth service provision has been internationally recommended for wound care for podiatrists, particularly through the pandemic [12], and has previously been trialled in Australia in rural and regional settings [30]. With limited knowledge about podiatrists’ common caseloads of musculoskeletal, paediatric or general care, it is difficult to determine the suitability of telehealth for these services. However, it may be reasonable to assume that telehealth was limited for high risk services in the private sector, due to low/no cost provision of service in public podiatry clinics around Australia. It is also unknown how many public hospital and community podiatry clinics maintained face-to-face care for high risk services. It may be reasonably assumed that high risk face-to-face services were maintained in most hospitals around Australia. Other professions in variable health settings described a rapid shift to the use of telemedicine with unknown effectiveness relating to condition-specific outcomes [31, 32]. Through the timeline of directives (Fig. 1), many of the major health insurance companies provided an item number for their customers to use to access telehealth by podiatrists in private settings, but there is limited knowledge of its uptake. Of note is that one of the largest health insurers removed this access after major restrictions were eased, citing low uptake by customers in accessing their podiatrist via telehealth [33].

A positive finding of this study was podiatrists reporting the use of reliable information sources such as jurisdictional governments and peak body information aiding business decision making. This may reflect the value placed on evidence informed sources by the responding podiatrists. Health professionals have been strongly urged to consider the spread of “fake news” throughout this time on social media and to take pro-active action against it [34]. While governments have a vested interest in the most accurate and detailed news being released, this places additional responsibility on peak bodies, such as the Australian Podiatry Association, to ensure accurate representation of these messages, due to frequency with which the professional seeks information from.

The quantitative data provides a picture of limited impact on SARS-CoV-2 on the podiatry profession, however the qualitative statements of impact described by respondents described the mental health impacts and resilience podiatrists have shown through rapid change. The challenges of increased likelihood of exposure or infection, fluctuating service delivery decisions, schooling at home, financial or job loss and increased responsibility have all been areas identified as placing increased stress on health workers in the past 10 months [35]. Podiatrists described positivity and assuredness in their skill mix for use of PPE, together with their flexibility in triage or lateral shifts in service delivery outside of the general podiatry skill set. Others appeared to instinctively employ techniques to reduce the impact on their staff, such as debriefing, regular updates and meetings. However, many also discussed that this came at a personal toll, similar to that seen in other countries and health care settings [36]. Development or exacerbation of mental health conditions, burnout or even development of post-traumatic stress disorders are prevalent in health care workers post pandemic responses. These have been documented subsequent to pandemic responses by both frontline and non-frontline health care workers during outbreaks of severe acute respiratory syndrome (SARS) or Middle East respiratory syndrome (MERS), regardless of the closeness of patient interactions or exposure to confirmed cases [37, 38]. This should urge all podiatrists, regardless of which country worked in, to be proactive in identifying and seeking support if they have ongoing health or mental health concerns during and subsequent to this time.

A number of research opportunities arise from these findings, but this research also has a number of limitations. Recent and setting-relevant workforce data is essential for health officials and government to make accurate recommendations and funding decisions. International workforce research groups could model similar survey design to understand internationally comparable data during the pandemic to understand and predict the impact of the pandemic on access to podiatrists to maintain foot health. Researchers should also consider collecting more detailed information on telehealth service provision (and for which health conditions, treatment modalities and their effectiveness), and more detailed information on case mix in each setting. While this data may be retrospective in nature, limiting burden to respond during a crisis, but provide valuable information in the future to aid and promote innovative service delivery. It is reasonable to consider the low survey response rate as being related to the challenging times when this survey was released. This means that while the data provides an accurate reflection of those who participated, it may not be reflective of the experiences of the entire podiatry profession. Lastly, there is little research on burnout, and mental health challenges in the podiatry profession, with only one study to date [39]. Future research should consider data collected prior to, and during this time for subsequent studies into the long-term impact of the pandemic on mental health in allied health.

## Conclusion

Podiatrists in Australia reported variable impact of the pandemic on their business decisions, limited impact on their PPE stores, and their valued sources of information. Podiatrists also described their “marathon” journey through the pandemic with quotes describing their challenges and highlights. Describing these experiences should provide key learnings for future workforce challenges, should further restrictions come into place. While the SARS-CoV-2 pandemic is still prevalent throughout the world, this study highlights the resilience of the profession and its adaptability during unprecedented times.

## Supplementary Information

**Additional file 1.**

## Data Availability

Request for further details of the data set and queries relating to data sharing arrangements may be submitted to Cylie Williams (cylie.williams@monash.edu). Aggregate or summarised data may be shared based on reasonable request.

## Abbreviations

SARS-CoV-2: Severe acute respiratory syndrome coronavirus 2
PAIGE: Podiatrists in Australia: Investigating Graduate Employment
IP: Internet Protocol
PPE: Personal protective equipment
IQR: Interquartile range
DHS: Department of Health Services
